# Impact of a Digital Atopic Dermatitis Educational Intervention on Hispanic Patients and Family Members

**DOI:** 10.3390/jcm12062130

**Published:** 2023-03-08

**Authors:** Luis Fernando Andrade, MaryJo Bekhash, Siri Choragudi, Juan M. Gonzalez, Rodrigo Valdes, Gil Yosipovitch

**Affiliations:** 1 Dr. Phillip Frost Department of Dermatology and Cutaneous Surgery, University of Miami Miller School of Medicine, Miami, FL 33136, USA; 2 School of Nursing and Health, University of Miami, Coral Gables, FL 33146, USA; 3 Department of Dermatology, University of Florida, Gainesville, FL 32610, USA

**Keywords:** atopic dermatitis, education, public health, racial disparities

## Abstract

With the increasing incidence of atopic dermatitis (AD) in the U.S., the highest prevalence of AD being found in Hispanic countries, and the rising Hispanic-American population, educational resources on eczema focused on Spanish-speaking populations are needed more than ever. As such, the primary goal of this project was to assess the beneficial impact of an educational intervention conducted through a virtual platform for Hispanic individuals living with atopic dermatitis. Utilizing WhatsApp, the study enrolled 55 participants diagnosed with AD and/or parents of children diagnosed with AD. Participants were enrolled in a seven-day educational module with daily topics on AD health knowledge. A health knowledge survey was administered before the educational program, upon completion of the program, and one month after completion. The program found a 14% increase in AD health knowledge upon completion of the program (*p* < 0.001). Most importantly, there was no significant difference found between the health knowledge survey submitted at program completion and one month after completion, signaling that health knowledge taught through the course was successfully retained by participants (*p* = 0.29). Qualitative themes involving atopic disease were additionally explored through group discussions, including mental health and peer stigma. This study is the first of its kind in dermatology utilizing the WhatsApp format. The success of retained health knowledge regarding AD demonstrates that future virtual endeavors can be effective and accessible methods of patient education overall for populations that might not have ease of access to major medical centers.

## 1. Introduction

According to the United States Census Bureau, the Hispanic population of the United States (US) has been steadily increasing each year. In 2017, people of Hispanic origin became the largest ethnic or racial minority in the US, making up 18.1% of the country’s total population. By the year 2050, the Hispanic population is predicted to reach nearly 100 million people, making up 26% of the US population [1]. The prevalence of atopic dermatitis in Hispanic adults living in the United States has been reported at 6.0% [2]. There has been a variation of AD prevalence reported among countries in Latin America, with countries such as Argentina having an overall prevalence of 9.7% and countries such as Brazil having an upwards of 20.1% prevalence [3] In the US, Hispanic people have faced several obstacles when accessing healthcare. For Hispanics with limited proficiency in English, communication is often limited, which makes it difficult for them to obtain and maintain longitudinal care with a healthcare provider [4]. Barriers to healthcare access, such as communication and language proficiency, are one of many factors driving health disparities in diseases prevalent in the Hispanic community, one of which is atopic dermatitis (AD).

Atopic dermatitis has a negative impact on the quality of life of patients and their families. The associated psychosocial morbidity of AD includes sleep disruption, depression, agitation, anxiety, altered eating habits, reduced self-esteem, and difficulty concentrating [5]. Patients with AD also report feeling embarrassed and stigmatized due to their skin lesions and have a more negative body image compared to healthy controls [6]. Of note, a previous study showed that mothers of children with AD had the same levels of stress as mothers of children suffering from other severe disabilities (e.g., profound deafness and insulin-dependent diabetes) [7].

Visits to medical settings for acute symptoms associated with atopic dermatitis are highest in the Hispanic population, specifically in the costly urgent care and emergency room settings [8]. Barriers to health access such as overall lower socioeconomic status, disparities in educational level, and denial of medication by insurance companies further exacerbate lower quality of life conditions in Hispanic individuals living with atopic dermatitis [8]. Of several factors identified with poor disease control of AD, Hispanic ethnicity was significant [9]. Similarly, Hispanic children presented with more chronic and recurrent symptoms associated with atopic dermatitis, even when factoring in socioeconomic disparities [10]. Hispanic children at all levels of management for atopic dermatitis were almost three times more likely than white children to visit a medical office due to symptoms of atopic dermatitis, even in groups categorized as having good management of disease control [11]. The previously mentioned social determinants of health, including Hispanic ethnicity and socioeconomic status, impact both patients and the health system negatively by increasing health utilization and worsening health outcomes in general.

As a public health intervention, much success has been seen in the usage of educational modules for positive quality-of-life outcomes in AD. A prior study using an educational website regarding AD health knowledge showed a significant improvement in the quality of life for participants both statistically and clinically [12]. Interventions that are culturally competent, cost-effective, easily accessed, and available in the Spanish language can provide a solution to the traditional barriers to health access found in the Hispanic community.

WhatsApp is a social media platform that emphasizes direct and group messaging. Compared to other applications such as Facebook and Instagram, WhatsApp remains the dominating messaging application in Hispanic culture, with surveys from Brazil revealing that over 93% of all smartphone users had the application downloaded on their phones [13]. Polling studies based in the US confirm this perspective as a recent National Tracking Poll (#201048) from New Morning Consult revealed that out of 999 Hispanic adults polled, nearly half admitted to having a WhatsApp account. In contrast, only 24% of adults overall mentioned usage of the app. This is a significant social and cultural phenomenon in the Hispanic community both internationally and in the U.S., where social media platforms such as Facebook and Instagram report the highest overall usage in the general population.

The social media application is both free and easy to access, with certain public health interventions taking advantage of this fact to develop educational interventions on the virtual platform. A recent educational intervention focused on breast cancer knowledge implemented the usage of WhatsApp to conduct lessons and found data in the post-survey showing significant increases in health literacy regarding risks, protective factors, and clinical manifestations of breast cancer [14]. Similarly, another study on diabetes control found a significant drop in HbA1c levels for the test group with the WhatsApp intervention compared to the control group that did not receive educational modules through the application [15]. No similar study in the current literature has been conducted for WhatsApp educational interventions targeting patients suffering from dermatological conditions.

A virtual platform for these interventions is a modern yet underutilized innovation that can overcome significant barriers to resource access such as cost, convenience, cultural understanding, and translation to the Spanish language. As such, this study developed a 1-week long WhatsApp-based educational intervention in Spanish focused on atopic dermatitis as a much-needed innovation for both the local Hispanic community and long-term reproducibility in Latin American countries with a high prevalence of atopic dermatitis.

## 2. Materials and Methods

Participants that identified as adults (aged 18 or older), living with a medical diagnosis of AD, and/or parents of children living with AD were recruited through a social media advertisement for the educational study, with additional inclusion criteria including access to a cellular device for the duration of the educational study, being Spanish speakers, and familiarity with the WhatsApp (Menlo Park, CA, USA) platform. Exclusion criteria included adults unable to consent, individuals under the age of 18, individuals unable to speak Spanish, individuals with severe hearing or visual impairment, and individuals unfamiliar with how to utilize the WhatsApp application. The main social media platform utilized for recruitment included virtual Facebook groups focused on patients living with atopic dermatitis sharing their lived experiences; IRB-approved text and flyer advertisements were posted in these virtual groups. A 25 USD gift card (Seattle, WA, USA) was offered as compensation for the completion of the program. Participants that expressed interest by commenting on the public post were contacted by the study team to obtain consent and enroll in the study. Enrolled participants completed a pre-survey on Qualtrics consisting of 37 true or false statements pertinent to atopic dermatitis health knowledge (Supplementary Materials). This questionnaire was developed by expert dermatologists in the field of atopic dermatitis and questions were made to evenly reflect and cover the material taught in each module. Questions were simplified to be able to be understood by participants at a middle school reading level. This same survey was administered to participants after the completion of the program and 1-month post-intervention completion. The educational intervention consisted of a weeklong module on a WhatsApp group chat led by a study moderator with daily educational modules consisting of educational text, audio messages, visual aids, and a summary video at the end of each day recapping the key takeaways from the daily module. Similar to the questionnaire, the educational module was created by experts in the field of atopic dermatitis and simplified to be accessible at a middle-school reading level. All documents were translated and back-translated by native Spanish speakers to provide accuracy in the translated format. New educational content was published each day in the group chat at a spread pace between 5:00 PM–10:00 PM EST. Discussion and questions were encouraged and moderated by the facilitator both during and outside of these hours. The study moderator was a research fellow on the study team with 3 years of experience in the clinical and research field of atopic dermatitis. Three separate educational groups were implemented to see if the group size has an impact on participation and outcome. Group 1 had 28 participants, Group 2 had 17 participants, and Group 3 had 10 participants join the study. This content was developed by experts in the field of atopic dermatitis. Participants were encouraged to ask questions and discuss among participants. Participants were prompted to reply to specific moderator questions throughout the daily lessons to track participation. Modules included an introduction to AD, up-to-date knowledge on the cause of AD, common triggers of AD, pharmacological and non-pharmacological treatments for AD, dietary considerations, and stress reduction/psychological impacts of AD.

Descriptive statistics of the surveys were summarized using means and standard deviations for continuous variables and proportions for categorical variables. The differences between the mean pre-survey, post-survey, and 1-month post-survey were determined by utilizing a one-way ANOVA. Two sample *t*-testing was performed to evaluate the difference in means between pre-survey vs. post-survey, and post-survey vs. 1-month post-survey. Mean difference and 95% confidence intervals were reported, and statistical analyses were conducted using SPSS version 27 (IBM, Chicago, IL, USA). The standard deviation of the response to individual survey questions was also examined to determine questions participants more frequently scored incorrectly.

Descriptive qualitative methodology was implemented by using standardized questions meant for individual responses by study participants to promote discussion. Qualitative statistics of the intervention were further collected using thematic analysis. Once the WhatsApp modules were finalized, transcripts were read from beginning to end, coded with themes, and evaluated for common summary points.

## 3. Results

Descriptive statistics are summarized in Table 1. Most participants in group 1 belonged to the 18–29 age category and the 30–39 age category. Most of the participants in the groups were females, had university-level education qualifications, and had a household income of less than 40,000 USD per year. A one-way ANOVA was performed to compare differences between the three groups on pre-survey and 1-month post-survey scoring. The analysis revealed there was no significant difference in health knowledge scoring between groups for both the initial survey (F(2, 52) = [2.932], *p* = 0.062) and the final survey (F(2, 31) = [0.022], *p* = 0.978). A one-way ANOVA was performed to compare the effect of the educational intervention on pre-survey, post-survey, and 1-month post-survey scoring. The analysis revealed there was a significant difference in health knowledge scoring between groups (F(2, 133) = [39.52], *p* < 0.001). A two-sample *t*-test was performed and revealed a significant increase (*p* < 0.001) in health knowledge between pre-survey (M = 26.51, SD = 3.27) and post-survey (M = 31.57, SD = 3.22). A two-sample t-test was performed and revealed no significant difference (*p* = 0.29) in health knowledge between post-survey (M = 31.57, SD = 3.22) and 1-month post-survey (M = 30.46, SD = 2.99). The percent increase in health knowledge from pre-intervention to post-intervention was calculated at 14%. The percent decrease in health knowledge from post-intervention to 1-month post-intervention was 3.2%.

The average health knowledge scores obtained by participants on the pre-survey, post-survey, and post-one-month survey are summarized in Table 2 and Table 3.

Standard deviations of individual survey questions were also examined to determine which questions were most frequently incorrectly answered to determine topics for future clinical education. The questions with the largest disparity in answer choices (SD = 0.45–0.50) were “Bleach and oatmeal baths are not helpful in treating eczema”, “Topical antihistamines work great for itch in atopic dermatitis”, “When applying the treatments on your skin, the back and front of your trunk require 6–7 fingertip units each of corticosteroid for eczema attacks”, “Children should eliminate foods considered ‘common allergens’ (eggs, wheat, soy) from their diets”, “Meditation and yoga are not helpful for symptoms of atopic dermatitis”, and “Wet wrap therapy can be used for periods longer than 2 weeks at a time”.

When examining socioeconomic factors, reported yearly income demonstrated a significant difference (*p* = 0.05) in the baseline knowledge score. Individuals reporting a yearly income of less than 40,000 USD, 40,001–60,000 USD, 60,001–100,000 USD and >100,000 USD had an average pre-survey knowledge percent correct of 71.7%, 70.5%, 83.8%, and 89.2%. respectively. There was a significant difference (*p* = 0.001) in the percent health knowledge increase from pre-survey to one-month post-survey across education level reporting. Middle school education, high school education, university education, and post-graduate education had a percent health knowledge increase of 17.6%, 14.14%, 10.76%, and 7.6%, respectively.

Qualitative data about the quality of living with atopic dermatitis were also collected through the module discussions. Topics and questions that garnished the most participation and discussion amongst the groups were coded by the facilitator to determine common themes. Common themes explored include symptoms of depression and reduced quality of mental health that were frequently reported by participants throughout the module. For parents of children living with eczema, bullying was a common theme throughout the schooling of children. Adults living with AD mentioned discrimination from co-workers, friends, and family in the form of ostracization through several assumptions about their disease status, including the misconception that the eczema flares were possibly contagious. Participants even reported stressful interactions with medical providers themselves, mentioning that often clinicians not well versed in the atopic dermatitis realm, underestimate the impact of pruritus on quality of life, and assume that the patients themselves are “exaggerating” or intentionally worsening their exacerbation through itching an otherwise mild exacerbation. Most participants in the intervention answered in the affirmative when inquired by the moderator about whether the material presented in each module was new to them. This revealed an additional common theme found throughout the educational module, which was the reported lack of exposure to educational AD health knowledge by prior health professionals, in particular for information regarding non-pharmacological treatment. During the educational lesson on non-pharmacological treatments, 70% and 90% of participant respondents mentioned they had never heard of wet-to-dry dressing therapy and bleach baths as treatment options for AD flares, respectively. A shared theme through both the anecdotes from parents of children with atopic disease and adults living with AD was a common misconception from peers that their disease has the potential to be contagious. The following is both exacerbated and potentially caused by the peer stigma surrounding the atopic rash: episodes of depression, anxiety, and low self-esteem. Participants reported incidents where they would selectively isolate themselves from social gatherings during episodes of exacerbations for fear of judgment from friends, families, and peers. Anecdotes of ostracization were reported both in school settings for children living with atopic dermatitis and the lack of employment opportunities in certain fields for adults living with the condition that was prone to frequent and visually evident exacerbations.

## 4. Discussion

To our knowledge, this is the first study in the field of dermatology utilizing the WhatsApp virtual platform to administer an educational intervention. The program found a 14% increase in AD health knowledge upon completion of the program (*p* < 0.001). Most importantly, there was no significant difference found between the health knowledge survey submitted at program completion and one month after completion, signaling that health knowledge taught through the course was successfully retained by participants (*p* = 0.29). As a whole, the results from our study demonstrate that the virtual WhatsApp platform can be an effective learning tool for patient education in addition to the standard of care offered to patients.

In addition to the increase in health knowledge, it is important to also recognize that the material learned through this 14% increase in health knowledge constitutes information that has the potential to cause a significant beneficial impact on the lives of people suffering from AD and their families. A theme of lack of knowledge regarding non-pharmacologic interventions was identified. While both bleach bath and wet-to-dry techniques are evidence-based practices that have been demonstrated to increase the quality of life and reduce itch exacerbation in patients living with atopic disease, our qualitative results showed that this was new material for a large proportion of participants. Not only were patients interested in learning more about at-home management of atopic dermatitis, but a portion of participants started actively implementing elements taught through the modules over the course of the week and providing feedback to their peers. The lack of familiarity with issues that otherwise might seem common knowledge by experts shows that there might be a lack of communication and patient education beyond medical management in the non-pharmacological treatment of eczema, or possibly even a lack of specialized education regarding atopic dermatitis and its treatment by health care providers not frequently exposed to the disease. Several participants who have their AD managed mainly by primary care providers (PCPs) commented that they, at times, feel the lack of clinician education and exposure to the more severe cases of AD can be impacting their overall outcomes.

In addition to receiving the educational material, participants were encouraged to discuss in the group setting and actively utilized the group chat setting to discuss how each of the modules was pertinent to life with eczema, paralleling a support group for people to confide in others living with similar experiences. Through group discussions, participants confided in each other with a common feeling of isolation in having atopic disease, and feeling, at best, a lack of empathy and understanding from members of their community regarding life with AD, and at worst, ostracization and discrimination because of their disease. One of the positive takeaways from these discussions on themes of depression and social isolation was that through the group format of WhatsApp, the participants found comfort in having the ability to discuss, support, and confide in other individuals that have lived through shared realities, something that a majority of participants have reported not having access to in their daily life.

Regarding WhatsApp functionality, patients commented that they had little to no difficulty accessing the educational modules through group chats, as WhatsApp is a common platform already utilized in South American countries and through Hispanic Americans living in the United States. Prior studies have shown that WhatsApp’s ease of access allows the utilization of the virtual platform for a large portion of the Spanish-speaking populace, especially elderly populations that might have difficulty utilizing newer applications such as Zoom [16]. Interestingly, the majority of study participants (69%) were in the 18–40 range, hinting that WhatsApp can be a preferential method of communication for Hispanic young adults as well compared to other virtual applications. In addition to the widespread use of WhatsApp in the Hispanic population, the benefits of this application include its speed and multimedia sharing capabilities including video, images, and audio in groups while encouraging collaborative discussion [17]. The asynchronous nature of a WhatsApp group setting also allows individuals working throughout the day to be able to review the course work at their own pace and be able to recap prior modules, videos, and audio clips through the streamlined chat settings.

While specialized patient education by experts in dermatitis at dedicated centers would be the gold standard of AD education, the availability, location of these centers, and language barriers can make this a difficult prospect for a lot of individuals, particularly Spanish speakers living in rural settings. With the advent of COVID-19, the pandemic brought to the forefront the realization that telehealth-based settings can be an effective method of health utilization both for individuals otherwise not able to physically access it and for ease of access to participants that would have otherwise had the means to attend in-person appointments. In addition to reaching a broader audience, a virtual intervention has the benefit of technically unlimited participant capacity that might otherwise be limited in physical settings. Additionally, as discussed earlier with the themes of peer ostracization for atopic disease, being able to discuss it in a private, virtual setting with peers might improve participation versus an in-person educational setting.

While all individuals were identified as Hispanic through the inclusion portion of the consent and enrollment process, only 83.6% of all participants selected ‘Hispanic’ in the social demographics of the survey, with 16% opting to select ‘White’ and/or ‘Other’. As the term Hispanic can encompass a wide variety of ethnic cultures and discussions on race and identity continue to evolve in the U.S., further studies regarding Hispanic acculturation or assimilation into the host culture, and its impact on atopic burden should be explored. For discussion purposes, our study has focused on the definition of Hispanic as both individuals with Spanish-speaking capacity and through the adopted National Institutes of Health definition as ‘A person descended from Spanish-speaking populations. People who identify their origin as Hispanic, Latino, or Spanish may be of any race.’ This distinction is important to highlight, as along with the previously mentioned differences in acculturation, ethnicity and language are not necessarily mutually exclusive, as shown by new generations of Hispanic-identifying Americans with less exposure to the Spanish language compared to Hispanic individuals living in predominately Spanish-speaking countries. This is a sociodemographic factor to consider for future public health studies.

One of the findings we discovered throughout the intervention was that the majority of respondents that expressed interest in the study (and eventually participated) were women. While statistical analysis revealed no significant difference between the pre-survey and post-survey health knowledge scores based on gender, the low power and skew towards female participants in this study may underestimate possible differences in educational intervention effectiveness across gender, as prior studies in the realm of atopic dermatitis have shown data supporting differences in AD treatment adherence among men and women [18].

## 5. Conclusions

Overall, this study demonstrated that the utilization of virtual interventions with elements embedded in a study population’s culture (e.g., common usage of WhatsApp in the Hispanic population) can be an effective public health intervention, particularly in the field of eczema education. Future studies can explore virtual interventions in other realms of dermatology, including other inflammatory skin diseases. Clinicians can utilize topics with the largest number of incorrect answers on the post-surveys, such as non-pharmacological interventions and technical application of topicals, to focus additional time on patient education. Furthermore, using this virtual intervention, we can extend beyond examining the knowledge base to assess the impact of educational interventions on quality-of-life measures, such as sleep, mood, and stress.

## Figures and Tables

**Table 1:** Sociodemographic variables.

Variables	Participants (*n* = 55)
	*n* (%)
Age (years)	
18–29	19 (34.5)
30–39	19 (34.5)
40–49	13 (23.6)
50–59	4 (7.4)
Gender	
Male	8 (14.5)
Female	47 (85.5)
Race	
White	5 (9.1)
Hispanic/Latino	46 (83.6)
Other	4 (7.3)
Highest level of education	
Primary	3 (5.5)
Middle school	4 (7.2)
High school	16 (29.1)
University	26 (47.3)
Postgraduate	6 (10.9)
Household income per year	
Less than USD 40,000	38 (69.2)
40,001–60,000 USD	11 (20.0)
60,001–100,000 USD	3 (5.4)
Over 100,000 USD	1 (1.8)
Missing	2 (3.6)

**Table 2:** Differences between the pre-survey score and post- survey score among all 55 participants.

Pre-Survey Score Mean (SD)	Post-Survey Score Mean (SD)	Mean Difference (95% CI) (Post-Survey Score—Pre-Survey Score)
26.5 (3.27)	31.6 (3.22)	5.1 (3.3, 6.9)

CI: confidence interval; SD: Standard deviation. Difference between mean scores were assessed using two samples *t*-test.

**Table 3:** Differences between the post-survey score and post-one-month-survey score among all 55 participants.

Post-Survey Score Mean (SD)	1 Month Post-Survey Score Mean (SD)	Mean Difference (95% CI) (Post-Survey Score—Pre-Survey Score)
31.6 (3.22)	30.4(2.99)	1.2 (−0.79, 3.19)

CI: confidence interval; SD: Standard deviation. Difference between mean scores were assessed using two samples *t*-test. No significant difference determined (*p* = 0.29).

## Data Availability

The data used to support the findings of this study are included in the article. Any additional data requests are advised to contact the corresponding author (luisandrade@med.miami.edu).

## Supplementary Materials

The following supporting information can be downloaded at: https://www.mdpi.com/article/10.3390/jcm12062130/s1, Open-ended questions intended to promote conversation.
