# Supplementary material for: Impact of a Digital Atopic Dermatitis Educational Intervention on Hispanic Patients and Family Members
Source: J Clin Med. 2023 Mar 8;12(6):2130. doi: 10.3390/jcm12062130 (PMC10057075; doi:10.3390/jcm12062130)
Supplement: Supplementary file 1 [file jcm-12-02130-s001.zip › jcm-2233463-supplementary.pdf]

TEXT: Direct text message on the WhatsApp Platform

AUDIO: Direct audio message on the WhatsApp Platform

VIDEO: Video file on the WhatsApp platform with visuals and audio

GRAPHIC: Visual graphics representing information

COLLAB: Open-ended question intended to promote conversation

FOLLOW-UP/COLLAB: Open-ended question intended to promote conversation and intended to gather answers to questions in the follow-up recap day

Day 2: What are the causes and triggers of atopic dermatitis?

What exactly is causing my Atopic Dermatitis?

[TEXT/VERBAL]: While experts are not too sure about the exact cause of the condition, physicians and scientists agree that both the condition itself and the attacks (sudden appearance or worsening of itch or rash) of eczema and itch are associated with the environment and our genes. While our own genes and the environment are things we can’t always avoid, recognizing certain environmental triggers that are avoidable can be useful in improving our quality of life when living with atopic dermatitis.

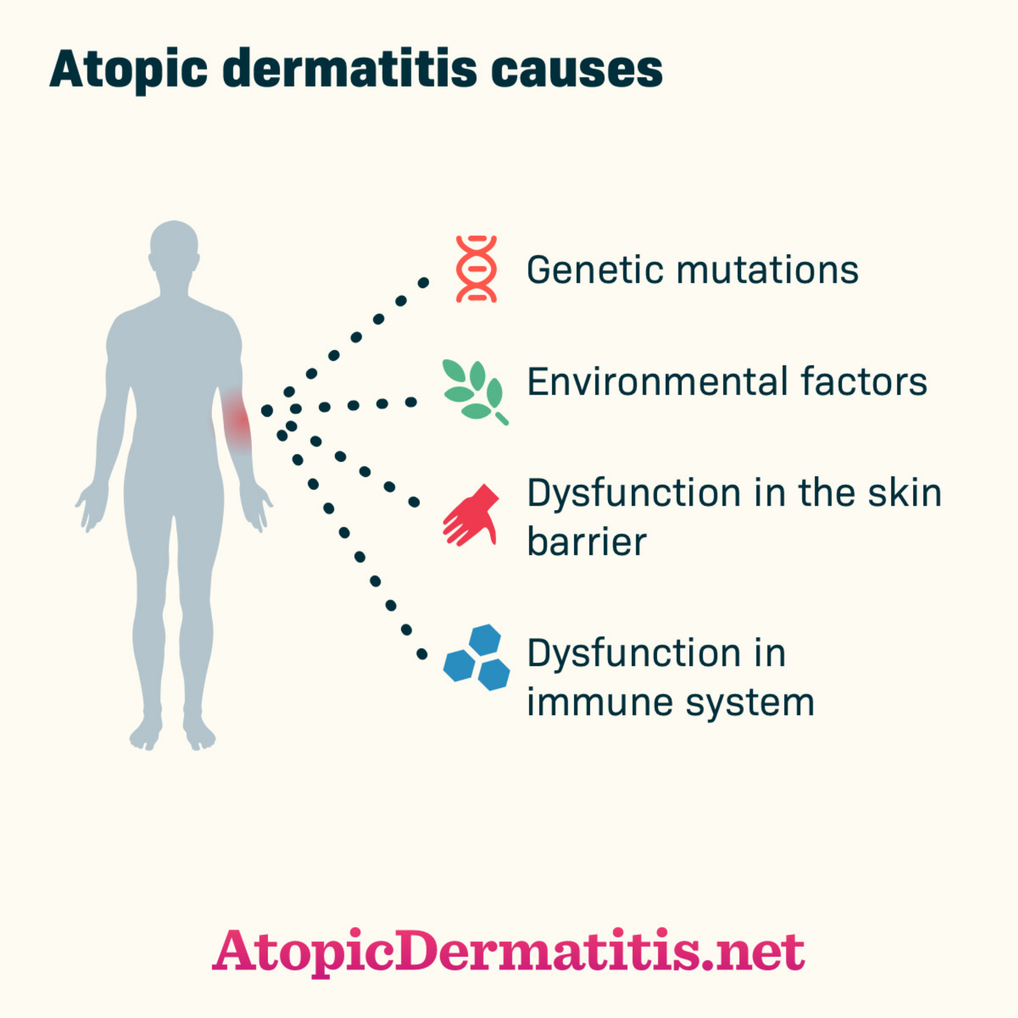

Can you tell me more about how experts believe genetics has played a role?

Our skin is our body's first line of defense against viruses and bacteria by having our skin cells hold tightly together to prevent the intruders from gaining access. Some individuals living with atopic dermatitis can have genetic factors that make their skin cells not hold as tightly as other individuals (e.g a deficiency in a protein that is present in living cells called filaggrin that helps make our skin strong), leading to “holes in the wall.” If you were to poke some holes in a water bottle, fluid would subsequently spill out. Similarly, viruses and bacteria now have easier access to “spill into” our bodies. This is what leads to inflammation and eczema attacks, along with the start of the itch-rash cycle, and a higher chance to get skin infections.

Studies have shown that over 70% of people with atopic dermatitis have a family history of AD, asthma, or allergic rhinitis, making genetics one of the strongest factors for the development of the disease.

What are some of the environmental triggers that can trigger eczema attacks?

Triggers differ from person to person. The trigger, the intensity, and how often we get **attacks** of eczema can all vary. Triggers can be caused by direct contact, food, and even stress itself.

What are some direct contact triggers I should keep an eye on?

Direct contact triggers include anything that can be considered chemically or physically hurtful to our skin. These products can lead to a worsening of atopic dermatitis. Examples of these include products such as body wash/soaps that may have cleaner ingredients that are overly drying or harsh. An example of active ingredients to avoid in your soap includes s**odium lauryl sulfate**, a chemical commonly found in foaming soaps. Products that contain chemical fragrances (e.g typically soaps and toiletries that advertise as having a certain smell) can also **trigger attacks of eczema** . Likewise, this similarly applies to the laundry detergents we use to wash our clothes. Overall, it is recommended to use gentle cleansers including hand soap, body wash, shampoo, and other products that are typically applied to the skin. The clothes we wear daily might have an impact as well; fabrics that mention “wool” or synthetics like “polyester and “nylon” can irritate the skin. Pure cotton and silk are some examples of clothing fabrics that are good alternative fabrics for individuals living with atopic dermatitis.

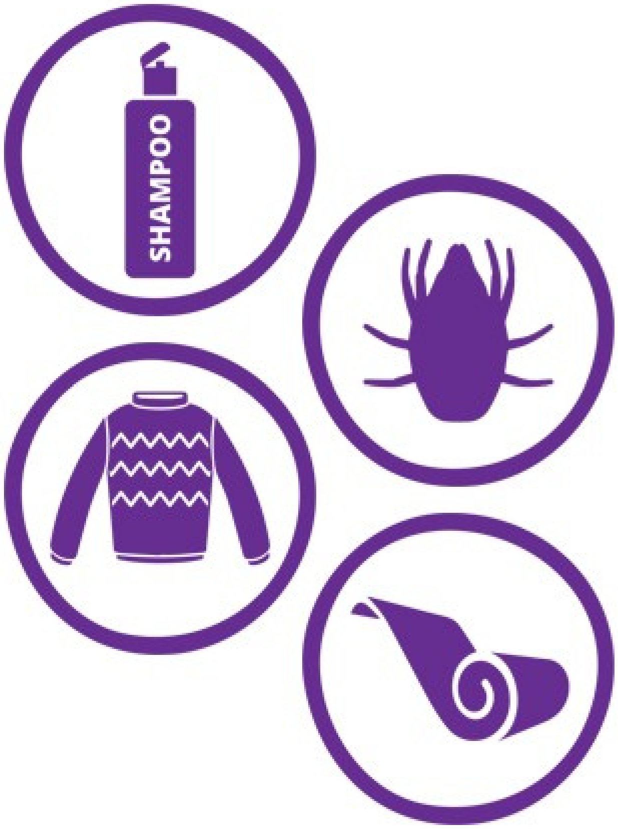

What are some food-related triggers for atopic dermatitis?

Several common allergies have been noted in individuals living with eczema: eggs, peanuts, soy, wheat, fish, cow's milk, and treenut-based products. Food-based allergies are typically more common in children living with atopic dermatitis compared to adults. We can determine food-based allergies safely by developing a plan of testing with your doctor. These tests can include food challenge testing followed by avoidance of foods that the test showed as possible triggers. However, there can be possible negative effects in elimination diets (e.g avoiding certain types of food) without the controlled supervision of a doctor. An example of this is the possible consequences of dietary and nutrition restriction in young children, particularly in dairy/cow milk products, due to its importance in growth rate and calcium intake. If food elimination diets are something a patient and/or their family is interested in pursuing, it is recommended to initially consult with a doctor and nutritionist to formulate a safe plan.

What are some psychological triggers for eczema?

Emotional triggers, such as stress, have been also associated with eczema attacks. It is no coincidence that for individuals living with atopic dermatitis that eczema attacks tend to become worse or increase in frequency right before a work deadline or an upcoming school exam. While the exact mechanism is not agreed upon, certain hypotheses include an increase of a hormone involved in stress that under stressful conditions can lead to further inflammation and attacks in an individual already living under constant inflammatory states; this further makes us more stressed which continues to worsen the eczema. This is a vicious cycle. Getting a good night's sleep, avoiding stressful situations, and when unavoidable, being able to control and implement relaxation techniques to improve our emotional wellbeing can be critical to maintaining a healthier baseline with reduced attacks and stress.

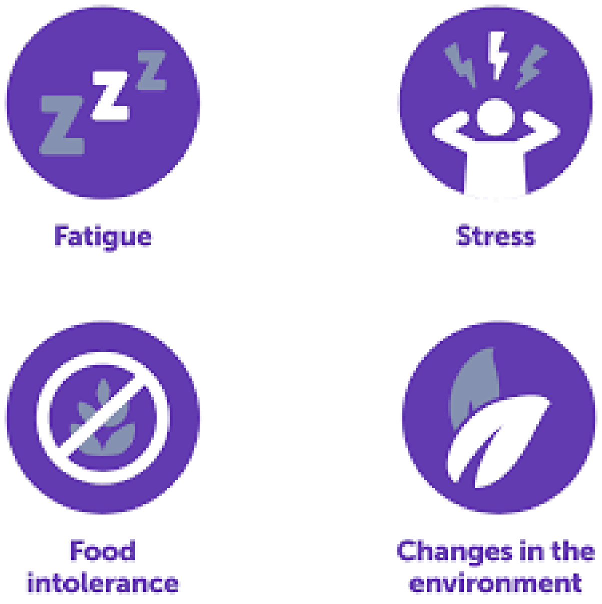

What are some methods I can “eczema-proof” my household?

While not all eczema triggers are avoidable, we can do our best to minimize the impact of triggers in our homes.

Dust and dust mites are common triggers for individuals living with atopic conditions such as eczema, and it is no surprise that a significant amount of dust can build up in our furniture over time. One of the easiest ways we can eczema proof our living quarters is with pillow sheets and bed-sheets that dust mite-proof. In addition, washing our sheets should be similar to washing our clothing; with gentle detergents, warm water, and products that have minimal ingredients that can cause irritation. Ingredients like fragrance (perfumes and additional ingredients meant to make products “smell good”) should be avoided.

r 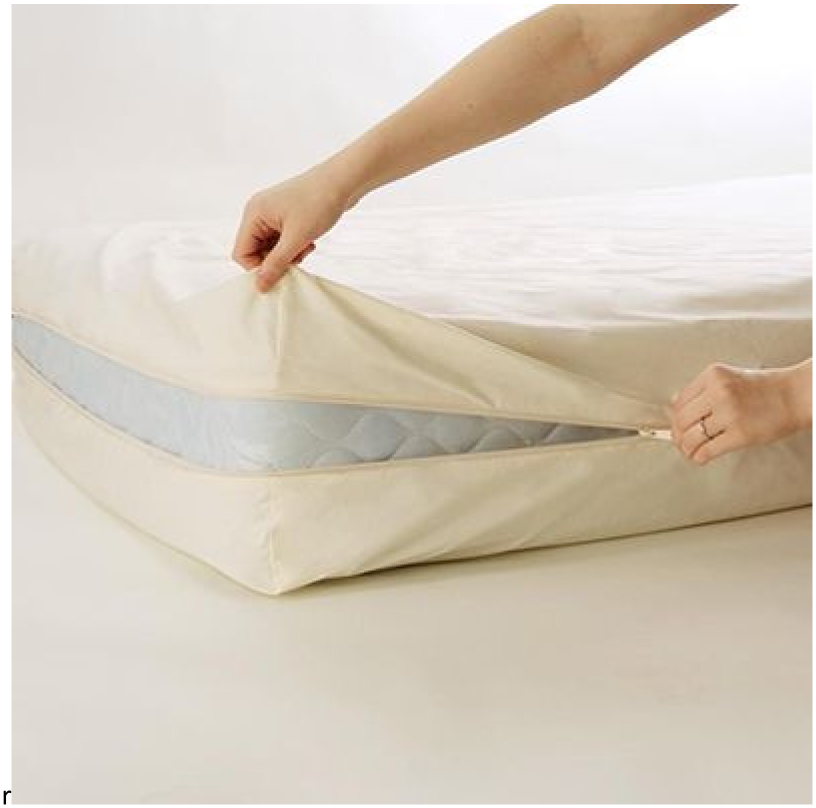

For our kitchen and bathrooms, it is important to have gentle cleansers to prevent drying out our skin. In addition, it might be beneficial to have some moisturizer right next to our soap dispensers so we can actively remember to moisturize our skin during circumstances where we have to do frequent washing, such as doing the dishes. We also recommend keeping a pair of cotton gloves to use around the house for general household chores; even doing laundry might cause some eczema triggers in sensitive hands. For activities such as washing the dishes, we can cover our cotton gloves with disposable vinyl or neoprene gloves. Aim to always implement lukewarm temperatures when having to expose water to your skin.

While it might be difficult for most to refurbish our homes with fabric that is more atopic friendly, we can still implement certain strategies to minimize dust exposure. To make our furniture more atopic dermatitis friendly, we can similarly use covers like the ones we implement in our bedrooms to reduce the buildup of dust-mites. Remember to vacuum and dust on a frequent basis.

Pets can often produce excess dander (e.g pet fur) that can trigger atopic dermatitis symptoms. Pets can be a vital part of the family for a significant percentage of people, and for individuals that chose to live with their furry friends and make contact unavoidable, setting boundaries such as keeping “pet-free zones” like your bed-room and the furniture can be a good way to minimize the impact of dander.

Sweat is a common trigger for people with eczema because it can dry out the skin and worsens the itch. Tips to eczema patients who exercise, which is the most common cause of sweat-induced itching, is to moisturize the skin before exercising, shower immediately after, and then moisturize your skin. Go for low impact workouts when you have eczema attacks, wear loose cotton clothing, remove sweat with a towel and drink a lot of water and avoid peak hours of sunshine

Sunscreen is important for all individuals, but especially those of us impacted by atopic dermatitis. Make sure to always have SPF 30 protection when outdoors, consistently reapply every two hours, and to use shade when in outdoor settings to prevent drying of our skin.

FOLLOW-UP/COLLAB: (Evening of Module 2) Credit Q’s

What are some eczema triggers that have impacted you and your family?

What have you learned from today's module?

VIDEO: Video file on the WhatsApp platform with visuals and audio

[Video Recap Posted on Tuesday Morning]

COLLAB: Open-ended question intended to promote conversation

What are some successful ways you have minimized eczema triggers?

What advice would you give others on effective ways to ‘eczema-proof’ your home?

What are some of the environmental triggers you find hardest to avoid?

Have you implemented any of the ‘eczema-proof’ home techniques? Which one’s worked? Which one’s didn’t?

Do food-related allergies impact your eczema, and how?

Does stress impact your eczema, and how?

Día 2: ¿Cuáles son las causas y los desencadenantes (des

·

en

·

ka

·

de

·

nan

·

tes) de la dermatitis atópica?

¿Qué está causando exactamente mi dermatitis atópica?

[TEXTO/VERBAL]: Auqnue los expertos no están muy seguros de la causa exacta de la dermatitis atopica, los médicos y científicos están de acuerdo en que tanto la afección en sí misma como los ataques (aparición repentina o empeoramiento de la picazón o sarpullido) estan asociados con la medio ambiente y nuestros genes. Aunque nuestros propios genes y el medio ambiente son cosas que no siempre podemos evitar, reconocer ciertos factores de riesgo ambientales que son evitables puede ser útil para mejorar nuestra calidad de vida cuando vivimos con dermatitis atópica.

¿Puede decirme más acerca de cómo los expertos creen que la genética ha jugado un papel?

Nuestra piel es la primera línea de defensa de nuestro cuerpo contra virus y bacterias al tener nuestras células de la piel unidas para evitar que los intrusos tengan acceso. Algunas personas que viven con dermatitis atópica pueden tener factores genéticos que hacen que las células de su piel no se mantengan tan apretadas como otras personas (por ejemplo, una deficiencia en una proteína que está presente en las células vivas llamada filagrina que ayuda a fortalecer nuestra piel), lo que lleva a "agujeros en la piel". la pared." Si hiciera algunos agujeros en una botella de agua, el líquido se derramaría posteriormente. Del mismo modo, los virus y las bacterias ahora tienen un acceso más fácil para "penetrar" en nuestros cuerpos. Esto es lo que conduce a los ataques de inflamación y eccema, junto con el inicio del ciclo de picazón y sarpullido, y una mayor probabilidad de contraer infecciones de la piel.

Los estudios han demostrado que más del 70 % de las personas con dermatitis atópica tienen antecedentes familiares de dermatitis atopica, asma o rinitis alérgica, lo que hace que la genética sea uno de los factores más importantes para el desarrollo de la enfermedad.

¿Cuáles son algunos de los desencadenantes ambientales que pueden desencadenar ataques de eczema?

Los desencadenantes difieren de una persona a otra. El desencadenante, la intensidad y la frecuencia con la que tenemos ataques de eczema pueden variar. Los desencadenantes pueden ser causados por el contacto directo, la comida e incluso el estrés mismo.

¿Cuáles son algunos desencadenantes de contacto directo que debo vigilar?

Los desencadenantes del contacto directo incluyen cualquier cosa que pueda considerarse química o físicamente dañina para nuestra piel. Estos productos pueden provocar un empeoramiento de la dermatitis atópica. Ejemplos de estos incluyen productos como gel de baño/jabones que pueden tener ingredientes limpiadores que se secan demasiado o son ásperos. Un ejemplo de ingredientes activos que debe evitar en su jabón incluye el lauril sulfato de sodio, un químico que se encuentra comúnmente en los jabones espumosos. Los productos que contienen fragancias químicas (por ejemplo, jabones y artículos de tocador que se anuncian con cierto olor) también pueden desencadenar ataques de eccema. Del mismo modo, esto se aplica de manera similar a los detergentes para ropa que usamos para lavar nuestra ropa. En general, se recomienda usar limpiadores suaves que incluyen jabón de manos, gel de baño, champú y otros productos que normalmente se aplican sobre la piel. La ropa que usamos a diario también podría tener un impacto; las telas que mencionan “lana” o sintéticas como “poliéster y “nylon” pueden irritar la piel. El algodón puro y la seda son algunos ejemplos de telas para ropa que son buenas alternativas para las personas que viven con dermatitis atópica.

¿Cuáles son algunos de los desencadenantes ambientales que pueden desencadenar ataques de eczema?

Los desencadenantes difieren de una persona a otra. El desencadenante, la intensidad y la frecuencia con la que tenemos ataques de eczema pueden variar. Los desencadenantes pueden ser causados por el contacto directo, la comida e incluso el estrés mismo.

.

¿Cuáles son algunos factores desencadenantes de la dermatitis atópica relacionados con los alimentos?

Se han observado varias alergias comunes en personas que viven con eczema: huevos, maní, soya, trigo, pescado, leche de vaca y productos a base de nueces. Las alergias a los alimentos suelen ser más comunes en los niños que viven con dermatitis atópica en comparación con los adultos. Podemos determinar las alergias alimentarias de forma segura si desarrollamos un plan de pruebas con su médico. Estas pruebas pueden incluir pruebas de desafío alimentario seguidas de la evitación de alimentos que la prueba mostró como posibles desencadenantes. Sin embargo, puede haber posibles efectos negativos en las dietas de eliminación (por ejemplo, evitar ciertos tipos de alimentos) sin la supervisión controlada de un médico. Un ejemplo de esto son las posibles consecuencias de la restricción dietética y nutricional en niños pequeños, particularmente en productos lácteos/de vaca, debido a su importancia en la tasa de crecimiento y la ingesta de calcio. Si las dietas de eliminación de alimentos son algo que un paciente y/o su familia están interesados en seguir, se recomienda consultar inicialmente con un médico y un nutricionista para formular un plan seguro.

¿Cuáles son algunos desencadenantes psicológicos del eczema?

Los desencadenantes emocionales, como el estrés, también se han asociado con los ataques de eczema. No es coincidencia que para las personas que viven con dermatitis atópica, los ataques de eczema tienden a empeorar o aumentar en frecuencia justo antes de una fecha límite de trabajo o un próximo examen escolar. Si bien no se acuerda el mecanismo exacto, ciertas hipótesis incluyen un aumento de una hormona involucrada en el estrés que, en condiciones estresantes, puede provocar más inflamación y ataques en un individuo que ya vive bajo estados inflamatorios constantes; esto nos hace aún más estresados, lo que continúa empeorando el eczema. Este es un ciclo vicioso. Dormir bien por la noche, evitar situaciones estresantes y, cuando sea inevitable, poder controlar e implementar técnicas de relajación para mejorar nuestro bienestar emocional puede ser fundamental para mantener una línea de base más saludable con ataques y estrés reducidos.

¿Cuáles son algunos métodos con los que puedo "proteger el eccema" de mi hogar?

Si bien no todos los desencadenantes del eczema son evitables, podemos hacer todo lo posible para minimizar el impacto de los desencadenantes en nuestros hogares.

El polvo y los ácaros del polvo son desencadenantes comunes para las personas que viven con afecciones atópicas como el eccema, y no sorprende que con el tiempo se acumule una cantidad significativa de polvo en nuestros muebles. Una de las maneras más fáciles en que podemos proteger nuestra vivienda contra el eccema es con sábanas de almohada y sábanas a prueba de ácaros. Además, lavar nuestras sábanas debe ser similar a lavar nuestra ropa; con detergentes suaves, agua tibia y productos que tengan un mínimo de ingredientes que puedan causar irritación. Deben evitarse ingredientes como fragancias (perfumes e ingredientes adicionales destinados a hacer que los productos “huelan bien”).

Para nuestra cocina y baños, es importante contar con limpiadores suaves para evitar que se seque nuestra piel. Además, podría ser beneficioso tener algo de humectante justo al lado de nuestros dispensadores de jabón para que podamos recordar activamente humectar nuestra piel en circunstancias en las que tenemos que lavarnos con frecuencia, como lavar los platos. También recomendamos mantener un par de guantes de algodón para usar en la casa para las tareas domésticas generales; incluso lavar la ropa puede causar algunos desencadenantes de eczema en manos sensibles. Para actividades como lavar los platos, podemos cubrir nuestros guantes de algodón con guantes desechables de vinilo o neopreno. Trate de implementar siempre temperaturas tibias cuando tenga que exponer el agua a su piel.

Si bien puede ser difícil para la mayoría renovar nuestros hogares con telas que sean más amigables con los atópicos, aún podemos implementar ciertas estrategias para minimizar la exposición al polvo. Para que nuestros muebles sean más amigables con la dermatitis atópica, podemos utilizar igualmente fundas como las que ponemos en nuestros dormitorios para reducir la acumulación de ácaros. Recuerde aspirar y desempolvar con frecuencia.

Las mascotas a menudo pueden producir un exceso de caspa (por ejemplo, pelo de mascota) que puede desencadenar síntomas de dermatitis atópica. Las mascotas pueden ser una parte vital de la familia para un porcentaje significativo de personas y para las personas que eligen vivir con sus amigos peludos y hacen que el contacto sea inevitable, estableciendo límites como mantener "zonas libres de mascotas" como su dormitorio y el Los muebles pueden ser una buena manera de minimizar el impacto de la caspa.

El sudor es un desencadenante común para las personas con eccema porque puede resecar la piel y empeorar la picazón. Los consejos para los pacientes con eczema que hacen ejercicio, que es la causa más común de la picazón inducida por el sudor, son hidratar la piel antes de hacer ejercicio, ducharse inmediatamente después y luego hidratar la piel. Realice ejercicios de bajo impacto cuando tenga ataques de eczema, use ropa suelta de algodón, quítese el sudor con una toalla y beba mucha agua y evite las horas pico de sol.

El protector solar es importante para todas las personas, pero especialmente para aquellos de nosotros afectados por la dermatitis atópica. Asegúrese de tener siempre protección SPF 30 cuando esté al aire libre, vuelva a aplicar constantemente cada dos horas y use sombra cuando esté al aire libre para evitar que se seque la piel.

¿Cuáles son algunos desencadenantes del eccema que le han afectado a usted y a su familia?

¿Qué has aprendido del módulo de hoy?

¿Cuáles son algunas formas exitosas en las que ha minimizado los desencadenantes del eczema?

¿Qué consejo le daría a otros sobre formas efectivas de 'a prueba de eczema' en su hogar?

¿Cuáles son algunos de los desencadenantes ambientales que le resultan más difíciles de evitar?

¿Has implementado alguna de las técnicas caseras a prueba de eczemas? ¿Cuál ha funcionado? ¿Cuál no?

¿Las alergias relacionadas con los alimentos afectan su eccema y cómo?

¿El estrés afecta su eccema y cómo?

Social Demographics

· Age (18-29, 30-39, 40-49, 50-59, 60 and over)

· Gender (Male, Female)

· Race/Ethnicity (Check All That Apply: White, Black, Asian, Pacific Islander, Native American, Hispanic/Latino, Other ___)

· Highest Level of Education Received (No schooling, Elementary, Middle, High, College, Graduate)

· Household Income (Less than $40,000, $40,001-$60,000, $60,001- $100,00, Over $100,000)

· Occupation

· Have you been diagnosed with eczema/atopic dermatitis?

· Are you the parent and/or caregiver of a child diagnosed with eczema/atopic dermatitis?

· Have you previously participated in an atopic dermatitis educational program (e.g eczema school, etc.)?

Itch Knowledge

1) The more people scratch, the more it can lead to itching.

True

False

2) Stress can make itching worse.

True

False

3) Using oral steroids regularly for a long time is a great way to treat eczema.

True

False

4) Using oral steroids regularly for a long time is the best way to treat severe itch in eczema.

True

False

5) Oral and topical medications targeting skin inflammation is the best way to treat severe itch in eczema.

True

False

6) Eczema affects the barrier provided by the skin.

True

False

7) Eczema is mainly caused by an allergy to foods.

True

False

8) Eczema can be treated with steroids taken by mouth or put on the skin.

True

False

9) Dust and dust mites are common triggers for individuals living with eczema

__True

False

10) Sweat can be a trigger for eczema attacks

True

False

11) Bath longer than 10 minutes provide a moisturizing effect on the skin

True

False

12) Detergents and soaps with fragrances can help with preventing eczema attacks

True

False

13) Wet wrap therapy can be done with moisturizers

True

False

14) Bleach and oatmeal baths are not helpful in treating eczema

True

False

15) We should moisturize after bathing or contacting water

True

False

16) Bar soaps are recommended versus liquid soaps

True

False

17) Topical antihistamines work great for itch in atopic dermatitis

True

False

18) When applying the treatments on your skin, one unit of topical corticosteroids is approximately the size of the tip of your index finger

True

False

19) When applying the treatments on your skin , the hand requires approximately 1 fingertip unit of corticosteroid for eczema attacks

True

False

20) When applying the treatments on your skin , the back and front of your trunk require 6-7 fingertip units each of corticosteroid for eczema attacks

True

False

21) Overall, biologic medications work by decreasing the inflammation in our bodies

True

False

22) All children with atopic dermatitis have food allergies

True

False

23) Children should eliminate foods considered ‘common allergens’ (eggs, wheat, soy) from their diets

True

False

24) A balanced diet involving vegetables, whole grains, healthy fats, and fish is the best diet for individuals with atopic dermatitis

True

False

25) Meditation and yoga are not helpful for symptoms of atopic dermatitis

True

False

26) Massage therapy for children can reduce symptoms of eczema attack and itch long-term

True

False

27.) Wet wrap therapy can be used for periods longer than 2 weeks at a time

True

False

28.) Wet wrap therapy includes applying the topical treatment to affected skin, covering skin with a wet layer, and covering the wet layer with dry fabric.

True

False

29.) Psychological treatments can be helpful for dealing with itch.

True

False

30.) Supplements, such as fish oil and vitamin D, have not been shown to improve symptoms of atopic dermatitis

True

False

31.) Hives, itchiness, or swelling are signs of a food allergy.

True

False

32.) Anaphylaxis is a life-threatening allergic reaction that may present with wheezing, swelling of tongue or throat.

True

False

33.) Skin or blood allergy testing is the best way to determine active food allergy.

True

False

34.) Wool, polyester, and nylon are clothing products that do not irritate the skin

True

False

35.) Pure cotton and silk are clothing products that do not irritate the skin.

True

False

36.) Steroids work by reducing an overactive immune system and decreasing swelling.

True

False

37.) Topical steroids should be avoided in areas with thinner skin, such as the face and skin folds

True

False

(1) Material de encuesta para pacientes

Demografía social

· Edad (18-29, 30-39, 40-49, 50-59, 60 y más)

· Género (Hombre, Mujer)

· Raza / Etnia (Marque todas las que correspondan: Blanco, Negro, Asiático, Isleño del Pacífico, Nativo Americano, Hispano / Latino, Otro ___)

· Nivel más alto de educación recibido (sin educación, primaria, media, secundaria, universidad, posgrado)

18-

· ¿Le han diagnosticado eccema/dermatitis atópica?

· ¿Es usted padre y/o cuidador de un niño diagnosticado con eccema/dermatitis atópica?

· Ingresos del hogar al ano (menos de $ 40,000, $ 40,001- $ 60,000, $ 60,001- $ 100,00, más de $ 100,000)

¿Ha participado anteriormente en un programa educativo sobre dermatitis atópica (por ejemplo, escuela sobre eccema, etc.)?

· Ocupación

Conocimiento de picazón

1) Cuantas más se rascan la piel, más puede provocar picazón.

Verdadero

Falso

2) El estrés puede empeorar la picazón.

Verdadero

Falso

3) El uso regular de esteroides orales durante mucho tiempo es una excelente manera de tratar el eczema.

Verdadero

Falso

4) El uso regular de esteroides orales durante mucho tiempo es la mejor manera de tratar la picazón severa en el eczema.

Verdadero

Falso

5) Los medicamentos orales y tópicos que se enfocan en la inflamación de la piel son la mejor manera de tratar la picazón severa en el eczema.

Verdadero

Falso

6) El eczema afecta la barrera proporcionada por la piel.

Verdadero

Falso

7) El eczema es causado principalmente por una alergia a los alimentos.

Verdadero

Falso

8) El eccema se puede tratar con esteroides orales o aplicados sobre la piel.

Verdadero

Falso

9) El polvo y los ácaros del polvo son desencadenantes comunes para las personas que viven con eccema

__Verdadero

Falso

10) El sudor puede ser un desencadenante de ataques de eczema

Verdadero

Falso

11) El baño de más de 10 minutos proporciona un efecto hidratante en la piel.

Verdadero

Falso

12) Los detergentes y jabones con fragancias pueden ayudar a prevenir los ataques de eccema

Verdadero

Falso

13) La terapia de envoltura húmeda se puede hacer con humectantes

Verdadero

Falso

14) Los baños de lejía y avena no son útiles para tratar el eczema

Verdadero

Falso

15) Debemos hidratarnos después de bañarnos o entrar en contacto con el agua

Verdadero

Falso

16) Se recomiendan los jabones en barra frente a los jabones líquidos

Verdadero

Falso

17) Los antihistamínicos tópicos funcionan muy bien para la picazón en la dermatitis atópica

Verdadero

Falso

18) Al aplicar los tratamientos en tu piel, una unidad de corticoides tópicos es aproximadamente del tamaño de la punta de tu dedo índice

Verdadero

Falso

19) Al aplicar los tratamientos en la piel, la mano requiere aproximadamente 1 unidad de corticosteroides en la yema del dedo para los ataques de eczema.

Verdadero

Falso

20) Al aplicar los tratamientos en la piel, la parte posterior y frontal del tronco requieren de 6 a 7 unidades de corticosteroides en la punta de los dedos para los ataques de eczema.

Verdadero

Falso

21) En general, los medicamentos biológicos funcionan al disminuir la inflamación en nuestros cuerpos

Verdadero

Falso

22) Todos los niños con dermatitis atópica tienen alergias alimentarias

Verdadero

Falso

23) Los niños deben eliminar los alimentos considerados "alérgenos comunes" (huevos, trigo, soja) de sus dietas

Verdadero

Falso

24) Una dieta equilibrada que incluya verduras, cereales integrales, grasas saludables y pescado es la mejor dieta para las personas con dermatitis atópica

Verdadero

Falso

25) La meditación y el yoga no son útiles para los síntomas de la dermatitis atópica

Verdadero

Falso

26) La terapia de masaje para niños puede reducir los síntomas del ataque de eccema y la picazón a largo plazo

Verdadero

Falso

27.) La terapia de envoltura húmeda se puede usar por períodos de más de 2 semanas a la vez

Verdadero

Falso

28.) La terapia de envoltura húmeda incluye aplicar el tratamiento tópico a la piel afectada, cubrir la piel con una capa húmeda y cubrir la capa húmeda con una tela seca.

Verdadero

Falso

29.) Los tratamientos psicológicos pueden ser útiles para tratar la picazón.

Verdadero

Falso

30.) No se ha demostrado que los suplementos, como el aceite de pescado y la vitamina D, mejoren los síntomas de la dermatitis atópica.

Verdadero

Falso

31.) La urticaria, la picazón o la hinchazón son signos de una alergia alimentaria.

Verdadero

Falso

32.) La anafilaxia es una reacción alérgica potencialmente mortal que puede presentarse con sibilancias, hinchazón de la lengua o la garganta.

Verdadero

Falso

33.) Las pruebas de alergia en la piel o la sangre son la mejor manera de determinar la alergia alimentaria activa.

Verdadero

Falso

34.) La lana, el poliéster y el nailon son prendas de vestir que no irritan la piel.

Verdadero

Falso

35.) El algodón puro y la seda son prendas de vestir que no irritan la piel.

Verdadero

Falso

36.) Los esteroides funcionan reduciendo un sistema inmunológico hiperactivo y disminuyendo la hinchazón.

Verdadero

Falso

37.) Se deben evitar los esteroides tópicos en áreas con piel más delgada, como la cara y los pliegues de la piel.

Verdadero

Falso
